# Prevalence in Potato of ‘*Candidatus* Arsenophonus Phytopathogenicus’ and ‘*Candidatus* Phytoplasma Solani’ and Their Transmission via Adult *Pentastiridius leporinus*

**DOI:** 10.3390/insects15040275

**Published:** 2024-04-15

**Authors:** André Rinklef, Sarah Christin Behrmann, David Löffler, Jan Erner, Martin Vincent Meyer, Christian Lang, Andreas Vilcinskas, Kwang-Zin Lee

**Affiliations:** 1 Fraunhofer Institute for Molecular Biology and Applied Ecology, Ohlebergsweg 12, D-35394 Giessen, Germany; andre.rinklef@ime.fraunhofer.de (A.R.); sarah.behrmann@ime.fraunhofer.de (S.C.B.); jan.erner@ime.fraunhofer.de (J.E.); martin.meyer@ime.fraunhofer.de (M.V.M.); andreas.vilcinskas@agrar.uni-giessen.de (A.V.); 2 Agrarservice Hesse Pfalz GmbH, Rathenaustrasse 10, D-67547 Worms, Germany; loeffler@ruebe.info (D.L.); lang@ruebe.info (C.L.); 3 Institute for Insect Biotechnology, Justus Liebig University of Giessen, Heinrich-Buff-Ring 26, D-35392 Giessen, Germany

**Keywords:** Cixiidae, Solanaceae, Hemiptera, proteobacteria, phytoplasma, bacterial tuber wilt

## Abstract

**Simple Summary:**

The planthopper *Pentastiridius leporinus* is the main vector of two bacterial pathogens (Arsenophonus and stolbur phytoplasma) that cause a disease known as syndrome basses richesses (SBR) in sugar beet, reducing the yield and sugar content. In 2022, *P. leporinus* nymphs were also found to transmit Arsenophonus in potato fields, causing symptoms like wilting, yellow leaves, and rubbery tubers. We monitored both pathogens in Southwest Germany in 2022 and 2023, revealing that *P. leporinus* adults can transmit Arsenophonus and stolbur to potatoes. The broad prevalence of Arsenophonus was maintained, whereas the prevalence of stolbur increased in most regions in 2023. Neither of the pathogens influenced the germination rate of potato tubers, and no abnormal growth was observed after germination. Arsenophonus was not detected in germinated shoots, but stolbur was present, emphasizing the need for plant material testing to prevent outbreaks of disease.

**Abstract:**

The planthopper *Pentastiridius leporinus* (Hempiptera: Cixiidae) is the main vector of two bacterial pathogens: the γ-proteobacterium ‘*Candidatus* Arsenophonus phytopathogenicus’ and the stolbur phytoplasma ‘*Candidatus* Phytoplasma solani’. These pathogens cause the disease syndrome basses richesses (SBR) in sugar beet (*Beta vulgaris*), which reduces the yields and sugar content. In 2022, potato (*Solanum tuberosum*) fields were found to be colonized by *P. leporinus,* and the transmission of Arsenophonus was confirmed, resulting in symptoms like wilting, yellow leaves, and rubbery tubers. We monitored both pathogens in Southwest Germany in 2022 and 2023. This revealed their widespread presence in potato tubers, although there were differences in regional prevalence. The broad prevalence of Arsenophonus was maintained in 2023, whereas the prevalence of stolbur increased in most locations. We confirmed that *P. leporinus* adults can transmit both pathogens to potatoes, but neither pathogen reduced the germination rate of tubers, and no plants showed abnormal growth after germination. Arsenophonus was not detected in germinated shoots, but 5.4% contained stolbur, emphasizing the need for plant material testing to maintain phytosanitary conditions.

## 1. Introduction

The planthopper *Pentastiridius leporinus* (Linné) (Hemiptera: Cixiidae) has been observed colonizing sugar beet (*Beta vulgaris*) since the early 1990s, and its life cycle is adapted to sugar beet crop rotations [1,2,3]. Adults lay their eggs on sugar beet plants, and the hatching nymphs feed on the roots of sugar beet and any subsequently sown cereals. The adults emerge in the following year and migrate to sugar beet fields with a lifespan reaching, in a few cases, over 30 days, where they deposit their eggs to complete the life cycle [4,5].

Adult *P. leporinus* are sap-sucking pests that vector two pathogens to sugar beet: the γ-proteobacterium ‘*Candidatus* Arsenophonus phytopathogenicus’ (hereafter described as Arsenophonus) and the stolbur phytoplasma ‘*Candidatus* Phytoplasma solani’ (hereafter described as stolbur) [6]. Arsenophonus infection causes syndrome basses richesses (SBR), which is characterized by yellowing older leaves, lancet-shaped leaf deformations, and vascular necrosis, reducing the sugar content of beetroots [6]. Stolbur was characterized by similar symptoms in France [2] but also causes rubbery tap root disease (RTD) in sugar beet in Southeast Europe, where different subgroups are present [7,8]. *P. leporinus* is the main SBR vector in Central Europe [9], whereas cixiids, like *Reptalus artemisiae* (Becker), formerly known as *Reptalus quinquecostatus* (Dufour), and *Hyalesthes obsoletus* Signoret, are RTD vectors in Southeast Europe [10,11]. The spread of both pathogens poses a threat to sugar beet growers in Central Europe (including Germany and Switzerland) and in Southeast Europe [7,12,13].

Stolbur also infects potato (*Solanum tuberosum*), causing reddening, the formation of aerial tubers, leaf discoloration, shortened internodes, and upward rolling of the top leaf, resulting in severe yield losses [14]. *H. obsoletus* and *Reptalus panzeri* (Löw) adults were identified as the main vectors for stolbur in potatoes [15,16]. However, *P. leporinus* nymphs were observed in 2022 colonizing potato fields in Southwest Germany, causing and completing their life cycle on potatoes [17]. Furthermore, Arsenophonus was detected in potato tubers taken from that region, with plants showing symptoms like wilting, yellow leaves, and rubbery tubers, and are referred to as bacterial tuber wilt. Subsequent transmission assays demonstrated the ability of *P. leporinus* nymphs and adults to transmit Arsenophonus to potatoes [17,18]. Although Arsenophonus can infect potato tubers, its propagation through infected seed tubers has not been verified. Moreover, we do not yet know the area of potato fields affected by Arsenophonus and its prevalence relative to stolbur. Here, we investigated the transmission of both pathogens by *P. leporinus* adults, with a focus on the preferred plant parts they infest and the presence of these pathogens in seed potatoes, allowing us to determine the transmission routes and prevalence of both pathogens in Southwest Germany.

## 2. Materials and Methods

### 2.1. Detection of Arsenophonus and Stolbur in Potato Fields

Potato samples were collected from August to October in Southwest Germany. Five plants with symptoms such as wilting, yellowing, and/or rubbery tubers were collected from each field. Three tubers from each plant were sectioned, and one slice containing the navel was freeze-dried and homogenized. The pathogens were detected as previously described [17]. Briefly, DNA was extracted from 0.075 g of homogenate using a modified CTAB method [19] and the pathogens were detected by TaqMan real-time PCR (qPCR) using Luna Universal qPCR Master Mix (New England Biolabs, Frankfurt, Germany), 25 ng of template DNA, and primer pairs KL437/438 and KL464/465 for the detection targeting the 16S rRNA region of Arsenophonus and stolbur, respectively [20]. The copy number was determined using absolute standard curves. Samples with a CT value > 33 were considered negative, which is equivalent to 29 copies for Arsenophonus and 17 copies for stolbur. In total, 20 plants without symptoms, in addition to symptomatic plants, were collected.

The prevalence of both pathogens was mapped for 2022 and 2023 using qGIS v3.28.1 (QGIS Association). Each plant was categorized as healthy (no pathogen detected), infected with Arsenophonus, with stolbur, or with both (double infection). Data from different fields were grouped into a region and expressed as a pie chart representing the prevalence of each pathogen relative to the sample size.

### 2.2. Germination Trials

We assessed the ability of 324 tubers to germinate following their collection from different fields in Southwest Germany. The tubers were analyzed by qPCR (Section 2.1) and planted in the greenhouse in Göttinger 7 × 7 × 8 cm pots at 20 °C with a 16 h photoperiod. The plants were watered frequently, and germination was monitored for four weeks. The effect of each pathogen on the germination rate was determined using a chi-squared test. After 11 weeks, the lower part of the shoot was cut from 59 germinated plants and DNA extracted from 0.05 g of dry biomass was used to detect the pathogens by qPCR (Section 2.1).

### 2.3. Transmission Assays

The ability of *P. leporinus* adults to transmit Arsenophonus and stolbur to potato plants was tested by releasing 10 field-collected adults (5 males and 5 females) per plant onto 20 potatoes and 6 sugar beet plants, followed by an inoculation access period (IAP) of 6 weeks. Six more potato plants were evaluated in the absence of planthoppers as a control group. Potato plants (variety Belana; Saatzucht Berding, Bockhorn, Germany) and sugar beet plants (variety Annarosa; KWS, Einbeck, Germany) were grown in Göttinger 3-L pots (Lamprecht-Verpackungen, Göttingen, Germany) filled with Frühstorfer potting soil LD 80 (Heinrichs, Ingelheim, Germany) and covered with expanded clay (Floragard, Oldenburg, Germany). Each plant was kept in a single Cavea PopUp size M net cage (Howitec, Bolsward, The Netherlands) and was watered three times per week. The temperature in the greenhouse chamber was set to 22/16 °C day/night with a 16 h photoperiod. At the beginning of the IAP, the potato and sugar beet plants were 30 days old. Planthoppers were collected on 21 June 2023 by netting in a sugar beet field in the south of Hesse (49.6885022, 8.4203155) and prior to inoculation identified according to [21].

Plants were collected for further analysis after the IAP and potato plants were divided into leaves, the upper-stem section (~20 cm above ground), the lower-stem section (ground level), and roots. Sugar beet plants were divided into leaves, lower stems, and beetroot tips. The plant parts were freeze-dried and homogenized, and 0.075 g of ground tissue was used for DNA extraction and qPCR (Section 2.1).

## 3. Results

### 3.1. Prevalence of Arsenophonus and Stolbur in Symptomatic Potato Tubers

**Figure 1:**
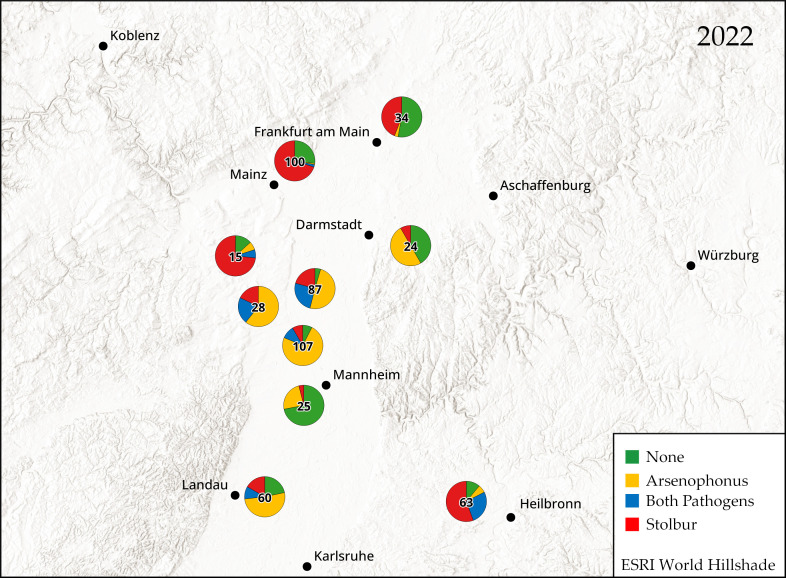
Prevalence of ‘*Candidatus* Arsenophonus phytopathogenicus’ (Arsenophonus) and ‘*Candidatus* Phytoplasma solani’ (stolbur) in symptomatic potato tubers (*n* shown in center) in 2022.

In 2022, Arsenophonus was detected in all the regions we investigated (Figure 1). In the Rhine Valley between Karlsruhe and Darmstadt, the prevalence of Arsenophonus was high in symptomatic plants, ranging from 24.0% to 84.1%. However, further to the north (north of Mainz) and to the west, the prevalence was lower (2.9–13.3%). East of Darmstadt, Arsenophonus was still detected in 13.3% of the samples. Further southeast in Baden-Württemberg, Arsenophonus was present in 33.3% of samples. In contrast, stolbur was the predominant pathogen in Baden-Württemberg and north of Mainz, with a prevalence of 44.1–82.5%. Stolbur was less prevalent in the Rhine Valley (4.0–46.0%).

**Figure 2:**
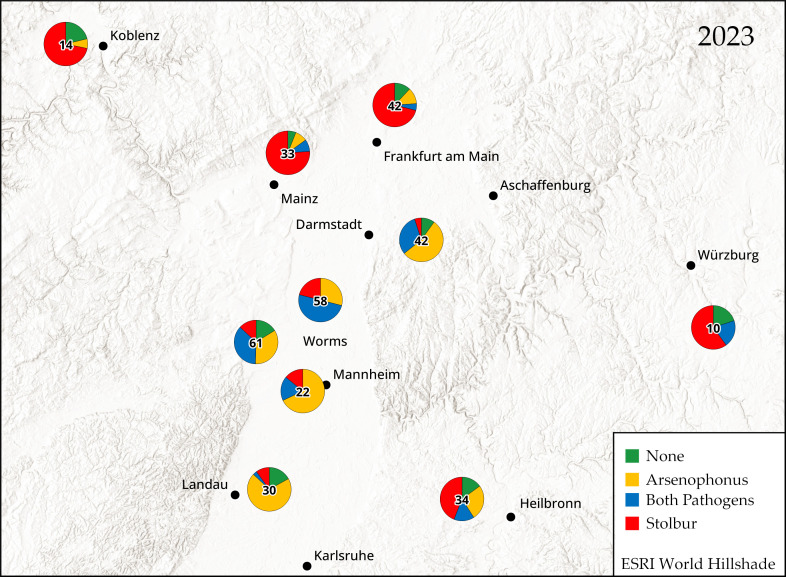
Prevalence of ‘*Candidatus* Arsenophonus phytopathogenicus’ (Arsenophonus) and ‘*Candidatus* Phytoplasma solani’ (stolbur) in symptomatic potato tubers (*n* shown in center) in 2023.

The overall prevalence of stolbur increased from 44.4% in 2022 to 56.4% in 2023 (Figure 2). Stolbur was the predominant pathogen in Baden-Württemberg, north of Mainz and Frankfurt, and south of Würzburg and Koblenz, with a prevalence of 58.8–84.8%. In the Rhine Valley, the prevalence was 13.3–70.7%. The overall prevalence of Arsenophonus was 47.9% in 2022 and 56.6% in 2023. As of 2022, Arsenophonus was predominantly found in the Rhine Valley, with a prevalence of 70.5–86.4%. In 2022, 18.7% of symptomatic plants showed no detection of any pathogens. However, by 2023, this percentage decreased to 10.4%. In only 1 out of 20 plants without symptoms, stolbur was detected, while Arsenophonus was not found.

### 3.2. Germination Assay

The germination rate of tubers with no pathogen present was 93.9% (Table 1). Tubers with only Arsenophonus present germinated at a rate of 92.8%, while tubers with stolbur only germinated at a rate of 86.1%. The germination rate of tubers with both pathogens present was 95.5%. These differences were not statistically significant with *p* = 0.212 (chi-squared), indicating that infection with neither pathogen inhibits the germination of potato tubers. In plants after germination, we observed no symptoms such as wilting, discoloration, and growth abnormalities in the infected plants or uninfected controls.

Following the germination of 37 stolbur-infected tubers, stolbur was detected in the stems of 2 of the resulting plants, representing an infection rate of 5.4% (Table 2). The CT values were 31.65 (44 copies) and 31.55 (46 copies), respectively. Arsenophonus was not detected in any of the 59 stems we tested.

### 3.3. Transmission Assay

Finally, we screened for the presence of Arsenophonus and stolbur in potato and sugar beet plants inoculated with *P. leporinus* (Table 3). Arsenophonus was detected in 13 of the 20 inoculated potato plants. In two of these cases, the pathogen was detected in the leaves, with a median copy number of 10,133. In seven cases, it was detected in the upper and lower shoot, with median copy numbers of 170 and 958, respectively. In the roots, it was detected in 6 out of 20 cases with a median copy number of 86. Stolbur was detected in 5 of the 20 inoculated potato plants. Notably, it was not detected in the leaves but was found in the upper shoots of 1 plant and the lower shoot of 2 plants, with median copy numbers of 45 and 6210, respectively. It was also detected in the roots of four plants, with a median copy number of 187.

Five of the six sugar beet plants were infected with Arsenophonus. In two plants, the pathogen was detected in the leaves with a median copy number of 129. It was detected in the lower stem and beetroot tip in every infected plant, with median copy numbers of 2684 and 5742, respectively. Stolbur was detected in three of the six plants. It was detected in the leaves of one plant (median copy number = 5918) and in the lower stem and beetroot tip of all three plants (median copy number = 182,076 and 78,061, respectively).

## 4. Discussion

Arsenophonus was detected in potato plants for the first time in Rhineland-Palatine and Hesse during the 2022 growing season and was present in 95% of tubers, but the extent of prior spreading was unknown [17]. We, therefore, monitored Arsenophonus in symptomatic potato plants at various locations in Southwest Germany and observed region-specific prevalence. However, stolbur-like disease symptoms were reported in the south of Hesse as early as 2020 [17], suggesting that Arsenophonus infections in these regions may have been misdiagnosed due to similar symptoms. Arsenophonus may have been present in potato fields much earlier, allowing the disease to spread without detection until 2022.

It was previously unclear whether the colonization of potatoes by *P. leporinus* and the subsequent transmission of Arsenophonus is a one-time event or the source of an annual recurrence. Our monitoring data indicate that the prevalence of Arsenophonus in Southwest Germany is comparable to that reported in our previous study [17], suggesting this pathogen poses a continuous threat to potato growers in these regions until effective control strategies are implemented.

We found that both pathogens were characterized by regional differences in prevalence. In 2022 and 2023, Arsenophonus was the predominant pathogen in the Rhine Valley, whereas stolbur was found predominantly around the periphery of this region. It is not yet clear why the observed symptoms are associated with regionally distinct ratios of both pathogens. Arsenophonus is found more frequently than stolbur in *P. leporinus* colonizing sugar beet [6,20,22]. The Rhine Valley has a high density of sugar beet fields [23], which may increase the likelihood of Arsenophonus transmission in these regions. In contrast, stolbur is more prevalent in regions with a lower density of sugar beet fields and, thus, a smaller population of *P. leporinus*, such that other vectors (like *H. obsoletus*) are likely to be relatively more abundant. In 2023, the prevalence of stolbur increased by 1.2-fold to 8-fold in all investigated regions except South Palatine and Baden-Württemberg. The precise reasons for this increase are unknown, but stolbur exhibits regional and seasonal variability in sugar beet and potato, with periodic outbreaks or epidemics [7,14,24]. In both years of observation, some plants showed symptoms, but no pathogen was detected. Given that the symptoms described for sample collection can be attributed to various other biotic and abiotic stresses, we expected a certain percentage of plants would show no detection of any pathogens. It may also be possible that Arsenophonus or Stolbur are present but have not yet reached the tubers.

Neither pathogen had a significant effect on the germination rate of potato tubers. Stolbur is known to upregulate callose synthesis in infected plants, forming filamentous stems upon germination [25,26]. We did not detect Arsenophonus in any germinated shoots despite a median copy number of 865 and up to 25,360 copies in the tubers, indicating no pathogen transmission via seed potatoes. This suggests *P. leporinus* is the sole mode of Arsenophonus transmission. In contrast, stolbur was detected after germination with a low copy number in the lower part of the stem. It is unclear whether this load is sufficient to induce systemic infection, enabling planthopper uptake and further transmission. Stolbur was previously reported to be present in 0.9–27.66% of germinated potato shoots [27]. Systemic infection may promote the spreading of stolbur to healthy plants during seed potato distribution, which would necessitate the testing of seed tubers to maintain phytosanitary conditions.

Arsenophonus and stolbur were recently shown to be transmitted to potato tubers by field-collected *P. leporinus* adults at rates of 48% and 12%, respectively [18]. By detection in the shoots, leaves, and roots, we observed higher transmission rates for both pathogens. Because stolbur and presumably Arsenophonus also typically follow the flow of assimilates from source to sink, overall transmission rates were higher by detection in the stem, through which both must pass to reach the tubers [28]. We detected the highest Arsenophonus titer in potato leaves. High copy numbers in source organs have already been reported for stolbur, due to similar modes of translocation [29]. However, the highest Arsenophonus detection rate was observed in the stems, and the highest median copy number was present in the lower shoot. For stolbur, the highest detection rate was observed in the roots, but the highest copy number was present in the lower shoot, indicating that the lower shoot is the most sensitive and suitable tissue for the testing of both pathogens.

In sugar beet, the detection rates were even higher for both pathogens. Arsenophonus was detected at the highest rate and with the highest copy number in the beetroot tip, whereas the highest copy number for stolbur was observed in the lower stem, making both parts suitable for pathogen detection. The median number of Arsenophonus copies in sugar beet stems was 2.8-fold higher than in potato, whereas the median number of stolbur copies was 29.3-fold higher than in potato, indicating that sugar beet is the preferred host for both pathogens.

## 5. Conclusions

We have confirmed that *P. leporinus* adults can transmit both Arsenophonus and stolbur to potato plants. However, planting out the infected potato tubers revealed no impact on the germination rate or time. Arsenophonus was not detected in stems after germination, but stolbur was present in 5.4% of stems derived from infected tubers. Further research is necessary to understand and quantify these transmission routes. Monitoring both pathogens in potato fields resulted in the widespread detection of Arsenophonus in Southwest Germany, indicating that colonization occurred earlier than 2022 when the pathogen was first detected in potatoes. In 2023, the prevalence of stolbur in potatoes had increased, whereas the prevalence of Arsenophonus was similar in 2022 and 2023. Our results highlight the need for effective control strategies against both pathogens and their vector *P. leporinus*.

## Figures and Tables

**Table 1:** Influence of ‘*Candidatus* Arsenophonus phytopathogenicus’ (Arsenophonus) and ‘*Candidatus* Phytoplasma solani’ (stolbur) infection on the germination of potato seed tubers. The *p*-value for the chi-squared test was 0.212.

	None	Arsenophonus	Stolbur	Both
Germinated plants	2/33	7/97	14/101	3/67
Germination rate	93.9%	92.8%	86.1%	95.5%

**Table 2:** Infection rate of potato plants following the detection of ‘*Candidatus* Arsenophonus phytopathogenicus’ (Arsenophonus) and ‘*Candidatus* Phytoplasma solani’ (Stolbur) in seed tubers.

	Arsenophonus		Stolbur	
	Negative	Positive	Negative	Positive
Infected stems	0/26	0/33	0/22	2/37
Infection rate	0%	0%	0%	5.4%
Median copies	-	-	-	45

**Table 3:** Detection rates and median copy numbers of ‘*Candidatus* Arsenophonus phytopathogenicus’ (Arsenophonus) and ‘*Candidatus* Phytoplasma solani’ (stolbur) in potato and sugar beet plants after inoculation with field-collected *P. leporinus* adults. Control potato plants were maintained in the absence of planthoppers. Note that the detection rates in different tissues do not add up to the overall detection rate because some plants were infected in multiple tissues.

Plant Species	Arsenophonus		Stolbur	
	Detection Rate	Median Copy Number	Detection Rate	Median Copy Number
Potato	65% (13/20)		25% (5/20)	
- Leaf	10% (2/20)	10,133	0% (0/20)	-
- Upper shoot	35% (7/20)	170	5% (1/20)	45
- Lower shoot	35% (7/20)	958	10% (2/20)	6210
- Roots	30% (6/20)	86	20% (4/20)	187
Sugar beet	83% (5/6)		50% (3/6)	
- Leaf	33% (2/6)	129	17% (1/6)	5918
- Lower stem	83% (5/6)	2684	50% (3/6)	182,076
- Beetroot tip	83% (5/6)	5742	50% (3/6)	78,061
Potato (control)	0% (0/6)		0% (0/6)	

## Data Availability

The data presented in this study are available on request from the corresponding author.
